# A European-wide exploratory study to analyse the relationship between training and energy efficiency in the construction sector

**DOI:** 10.1007/s10669-022-09891-x

**Published:** 2023-01-15

**Authors:** Irini Barbero, Yacine Rezgui, Ioan Petri

**Affiliations:** School of Engineering, Cardiff University, Cardiff, UK

**Keywords:** Training, Energy efficiency, Skills, Construction sector

## Abstract

Current evidence that supports the correlation between training and energy efficiency in the construction industry is sparse and lacks an in-depth and sector-wide analysis. Several context-specific (in terms of application, workforce segment, and scope) studies have highlighted several barriers, challenges, and gaps in the training landscape in the European construction sector. However, these do not scale up and translate to robust evidence for the entire industry. The paper aims to address this gap by adopting a quantitative and qualitative Europe-wide consultation that not only seeks to gather evidence about the relationship between training and energy efficiency but also broadens the scope of the investigation beyond this aim to understand the complexity of the training landscape in energy efficiency and to provide context to the resulting evidence, in a way that promotes generalisation of the results. A mixed-method approach is adopted involving secondary (in the form of industry studies and academic publications) and primary sources of evidence. The latter include a questionnaire (n = 52), a series of interviews (n = 28), an expert workshop, and use cases drawn across Europe providing examples of the correlation between training and energy efficiency. Five key themes emerged from the consultation, namely: (a) lack of systematic process to codify best practice into re-usable knowledge, (b) lack of industry-wide shared vision, (c) nature of the training available in the energy efficiency domain, (d) level of reliance on a trained and skilled workforce in energy efficiency, (e) efficiency of legislative frameworks, policies, and government incentives. While the analysis of the results confirms the correlation between training and energy efficiency, further efforts are needed to establish robust quantitative evidence. The research also points to several policy measures, including the need for adapted instruments to promote mutual recognition of energy skills and qualifications in the European construction sector.

## Introduction

The United Nations Environment Programme (UNEP) emphasized the imperative for all nations to pursue efforts to drastically reduce GHG emissions (UNEP 2021). It also called for a green Covid-19 pandemic recovery with more ambitious net-zero commitments. The pre-COP26 measures agreed during the 2015 Paris Agreement on climate change (IPCC 2018) would have only reduced predicted 2030 emissions by 7.5%; whereas reductions of 30% are needed to stay on the least-cost pathway for 2 °C and 55% for 1.5 °C (IPCC 2021; Cohen et al. 2021). There is a pressing need for every sector in industry, including the entire building and construction supply chain, to decarbonise by 2050. In this context, the construction industry is faced with the challenge and opportunity to reduce energy demand, improve process efficiency, and reduce carbon emissions (Rezgui and Miles 2010; Alreshidi et al. 2018; Li et al. 2019).

Energy efficiency demands adapted technology solutions, strategies (including training and education), and policy-making approaches that should be embraced by the entire supply chain across the whole lifecycle of a project (Hodorog et al. 2021; Sparrevik et al. 2021; Alhamami et al. 2020). One interesting example is the Energy Performance in Buildings Directive (EPBD 2018), which defines the scheme for Energy Performance Certification. The related energy audits, energy management systems, and energy manager/assessor training and certification are awareness programs that are usually effective in promoting energy efficiency and increasing the demand for a skilled workforce (Li et al. 2019).

The paper argues that the overall potential for energy efficiency would be higher if successful training initiatives and supporting policy instruments are put in place. In fact, staff training initiatives tend to be relatively low-cost activities and have been demonstrated to have large positive effects on the promotion of energy efficiency in industry (Backlund et al. 2012; Maier et al. 2019). As such, educational (both initial university curriculums and vocational education and training) and informative programs are ideal pathways to maximize demand for sustainable energy skills in the Construction sector. However, current evidence that supports the correlation between training and energy efficiency in the construction industry is sparse and lacks an in-depth and sector-wide analysis. The paper aims to address this gap by adopting a quantitative and qualitative Europe-wide consultation that not only seeks to gather evidence about the relationship between training and energy efficiency but also broadens the scope of the investigation beyond this aim to understand the complexity of the training landscape in energy efficiency and to provide context to the resulting evidence, in a way that promotes generalisation of the results.

Following this introduction, Sect. 2 summarizes related work. This is followed by a description of the methodology that underpins the research. Section 4, 5, 6 and 7 summarize the results of the various instruments used in the research, including the questionnaire, interviews, best practice use cases, and virtual workshop, respectively. These results are discussed in Sect. 8. Finally, the paper provides concluding remarks and directions for future research.

## Related work

Energy efficiency demands adapted technology solutions, strategies (including training and education), and policy-making approaches that should be embraced by the entire supply chain across the whole lifecycle of a project (Barbero et al 2022). In order to achieve the energy consumption and carbon emission reduction phased targets, there is a need to reconsider the role of socio-technical factors in the industry, including training and education (Hodorog et al. 2020). As Chai and Yeo argue: “All too often, the issue of climate change is treated as a purely technical one, outside the realm of social sciences or education unless to raise awareness. […] A vital element in this transition is an energy literate labour force equipped with the knowledge, skills, and competences (KSCs) to carry out the work”. Training has also been considered to facilitate the effectiveness of the integration of the necessary measures (Garmston and Pan 2013). Within the context of the EEB project, which included six geographical regions (Brazil, China, EU, India, Japan, USA), training was identified as a key driver towards better implementation of energy efficiency, in the building sector (Aerschot et al. 2009). More recently, and on EU level, the BUILD UP Skills highlighted the correlation between demand for energy efficiency and the need to train white-collar and blue-collar workers in the construction sector (European Commission 2016). However, the industry presents a fragmented landscape with several challenges (Barbero et al 2022). As highlighted by BUILD UP skills (Build Up 2020), these could be summarised as follows: ‘Economic barriers (lack of time for training, cost of training), awareness-related barriers (lack of understanding of the importance of skilled / trained workers), legal barriers (delays in introducing energy efficiency-related definitions), market barriers (low demand for energy efficient buildings and thus for the skills required to build them), and knowledge barriers (language, varying levels of competence of the trainees, and lack of facilities for practical training)’ (European Commission 2018). The digitalisation of the education and learning sector is paving the way to new changes in the form of (a) provision of smart content, (b) differentiated and personalized learning, (c) global and remote learning, and (d) administrative efficiencies (Marks et al. 2021). It has also been demonstrated that online learning outcomes do not differ significantly from traditional classroom education (Pyzhova et al. 2022). Conversely, the digitalisation of our economy and industrial sectors is affecting the demand for learning and education (Sony and Mekoth 2022; Palazzeschi et al. 2018). Industrial processes are constantly changing, prompting the need for employees to engage in active learning, training, and education (Bode et al. 2018; Störmer et al. 2014). As an example, the recent adoption of building information modelling (BIM), and the quest to decarbonise our built environment, have impacted several segments of the supply chain, including design, and engineering practitioners, prompting the need to redefine the construction personnel roles along with associated skills and competencies (Hodorog et al. 2021).

In this context, employees are increasingly feeling the pressure to continuously train, educate and retrain (Wulfken and Müller 2017) to remain competitive and increase their adaptability potential to the job market (Sony and Naik 2020). Consequently, employees will have to continuously unlearn, learn, train, and educate themselves in several areas related to their core expertise (Schallock et al. 2018), in an autonomous and self-organised manner (Adam et al. 2019; Sony and Naik 2020). It is interesting to note that as the imparted training changes, the workforce will begin to divide into an older cohort which received ‘traditional’ training and a younger cohort which received ‘future-thinking’ training. This dichotomy presents both intergenerational training opportunities and management challenges in organising a workforce with a non-uniform core skill set (Bergsagel and Isaac 2022).

The literature points to the importance, as well as the promising value, of training for energy efficiency in the construction sector for achieving national and wider energy targets (Brown et al. 2017; Backlund et al. 2012). Succar and Sher (2014) analysed the way in which organisational and educational institutions have started to adapt their delivery systems to meet changing demands on the market. Hodorog et al. (2021) adopted a novel text-mining approach which analyses social media alongside secondary sources of evidence to establish a level of correlation between BIM roles and skills to inform training and educational programmes. The authors have also evidenced that (a) construction skills and roles are dynamic in nature and evolve over time, reflecting the digital transformation of the Construction industry, and (b) the importance of socio-organisational aspects in construction skills and related training provision.

It is worth noting the Strategic Energy Technology (SET) Plan Roadmap on Education and Training, which has set the groundwork for the implementation of skilled workers towards innovations in the field of energy technologies (Maier et al. 2019). Furthermore, the BUILD UP Skills initiative, which specifically investigated the education and professional development of craftsmen and other on-site construction workers (BUILD UP 2020) from 28 Member States in Europe offers a significant blueprint for future actions. It has been suggested that an increase in demand will increase the need for professional development and upskill of both white and blue-collar workers, and that, overall, more concrete action needs to be taken in that direction (European Commission 2016). The European Qualifications Framework (EQF) has been a driving force in coordinating the efforts for qualifications of training across Europe. Drawing on previous relevant efforts, such as NQF (Bohlinger 2019), EQF has been developing steadily in the last decade.

Several context-specific studies have provided insight on the link between training and energy efficiency, by highlighting barriers, challenges, and gaps that are currently present in the construction industry (Preziosi et al. 2022). However, more evidence and further efforts are needed through which to investigate the nuances, causality, and dynamics of this relation. It is of high importance to investigate the fragmented landscape of training for energy efficiency for both blue-collar and white-collar workers, to be able to formulate conclusions and suggestions for future actions. This paper addresses the above gaps, building on previous findings and insights, towards the intensification of efforts for achieving concrete action for energy efficiency in the construction sector.

## Methodology

The general aim of this study is to establish the relationship between training and energy efficiency. As such, the research addresses the three following research questions:

RQ1: What is the state of awareness, access to information, and dissemination of knowledge for energy efficiency in the Construction sector?
RQ2: What is the level of provision of energy efficiency training in the Construction sector (in terms of scope, quality, content, cost, etc.)?
RQ3: Does training translate into effective sustainable and energy efficient interventions?

**Fig. 1:**
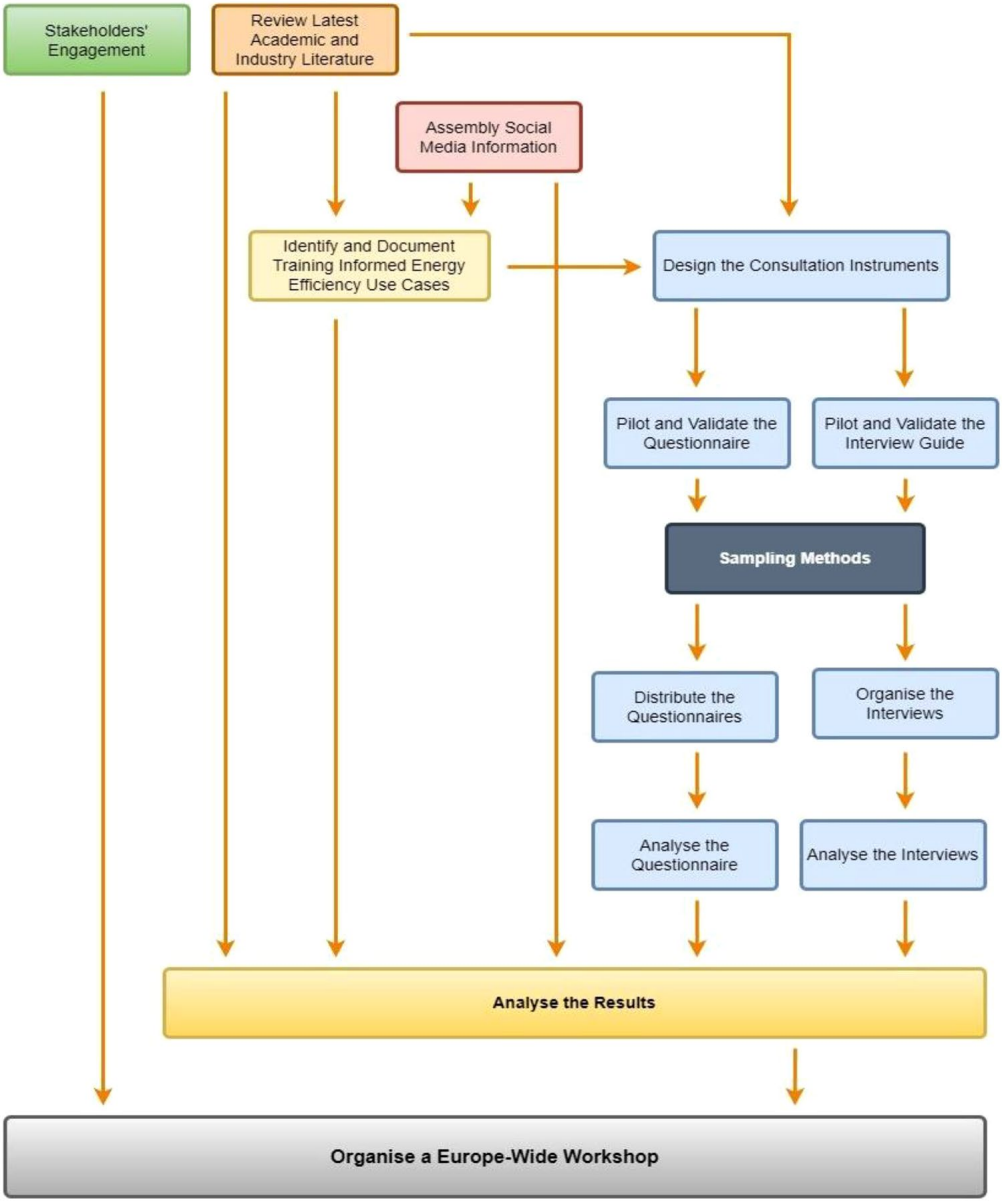
Illustrated methodological plan and process, with detailed steps

The study targeted the whole supply chain, including blue and white collars, across the project lifecycle, from inception to operation (in-use). The research followed a mixed-methods approach which includes quantitative and qualitative sources of evidence. This approach integrates several steps as illustrated in Fig. 1.

The study relies on both quantitative and qualitative data. It employs a mixed-methods approach, which is linked to a broader pragmatic theoretical framework (Descombe 2014). The design of the instruments (interviews and questionnaires) was formulated by addressing gaps and challenges evidenced by the literature. The questionnaires consist of a blend of multiple-choice questions and in some cases questions with options to further elaborate, so as to elicit comprehensive insights, as much as possible. For the interviews, the format was semi-structured, and the questions were open-ended. In order to test the consultation instruments (questionnaire & interview), the material was first disseminated among the Cardiff University research team. After the first round of feedback, the material was piloted through the wider network of the INSTRUCT project's consortium. This process allowed the appropriate corrective actions to take place before the consultation material was released for the data collection. Twenty-eight interviews were conducted and were analysed in NVivo. The pool of participants was formulated by nominating local stakeholders via the INSTRUCT project European network, in several European countries. The participants were, in their majority, white-collar workers, which is considered a limitation of the study. The interviews were conducted online, due to the COVID-19 pandemic and geographical limitations. The questionnaires were distributed to the INSTRUCT project network of partners, using a snowball sampling method, via SurveyMonkey. Fifty-two responses were collected, and the data were analysed via the SurveyMonkey's software and visualised with diagrams. A Europe-wide workshop was held on an online platform, involving fifteen participants from eight European countries. There were no blue-collar representatives, which is a limitation of the research. However, several of the workshop participants had experience of working with blue-collar workers. The aim of the workshop was to brainstorm and ask participants to comment and provide feedback on the five themes of the research. The use case collection was facilitated with the collaboration of experts who were asked to register a list of authoritative URI sources, registered at the http://www.energy-education.com platform. They follow the following categorisation: Objective-based analysis, Use-case type analysis, Building type analysis, Project type analysis, Target discipline analysis, Lifecycle stage analysis, and Impact-based analysis. For the assembly and analysis of social media information, the following steps were followed: Step 1: Identification of authoritative social media (twitter) accounts (endpoints). Step 2: Mining of social media accounts and extraction of knowledge related to the study, including roles, skills, trends for energy-education. Step 3: Inclusion of the outcome in the study. The list of the organisations utilised for the social media data gathering process is obtained from three sources: forensics algorithms for IP detection and organisation identification, followers of the selected social media (twitter) account, and identified training institutions (Hodorog et al. 2020).

For the questionnaire, the data were collected using SurveyMonkey software. The results were analysed along three categories: (a) Demographic, (b) Correlation between training and energy efficiency, and (c) Provision and content of training for energy efficiency.

For the interview analysis, an overall interpretive and qualitative approach was followed, by conducting a content analysis of the transcripts of the interviews (Vaismoradi et al. 2019). The aim was to extract, measure and interpret common emerging themes, which were categorised into nodes in NVivo. For each question, depending on whether it was an open-ended or close ended question, the respective nodes were created and then interpreted. The results of the analysis were also illustrated in diagrams.

Throughout the process of this study, the researchers were sensitive to biases by being aware that there are multiple interpretations of reality (Merriam 1988), thus prompting the use of an interpretive philosophical stance. The overall data collection period extended from July 2021 through December 2021, during which time (a) secondary sources of evidence were gathered through the literature review, (b) questionnaires were distributed after a pre-testing stage, (c) interviews were conducted after a pre-testing stage, (d) best practice use cases were collected, and a workshop was held. All ethical approvals to conduct the research were obtained in advance. All participants were informed of their right to decline to participate in this study. They were also informed of their right to anonymity. The nature, purpose, and objective of the study were clearly explained to every participant. As stressed by Bonoma (1985), collecting different types of data by different methods, from different sources, produces a wider scope of coverage and results in a fuller picture of the phenomena under study than would not have been achieved otherwise. Problems of construct validity have been addressed using the variety of sources of information described above. The development of converging lines of inquiry in this manner is known as ‘‘triangulation’’ and is considered as a process of using multiple perceptions to clarify meaning and assess the validity of an interpretation (Stake 1995).

## Questionnaire results

A total of fifty-two validated (i.e., complete) responses were collected, out of a total of 120 distributed questionnaires (representing a 43% response rate) targeting different age groups and professionals across the entire construction value chain. 57.58% of the respondents were male and 42.42% female. The respondents’ distribution across disciplines is illustrated in Table 1. It is worth noting that out of the targeted respondents a large majority were White Collars, while only Contractors and subcontractors represented Blue Collars. By white collars, the authors mean professionals who do not perform manual work.

**Table 1:** Fields of expertise of participants

Expertise	Numbers
Fire engineering	1
Briefing	4
Electrical engineering	5
Facility management	5
Architectural engineering	6
Mechanical engineering	9
Structural/civil engineering	10
Architectural design	11
Other	15
Project management	21

When asked about the barriers to training in the industry (Table 2), “Financial / funding issues” (50.00%) and “Not enough time for training” (46.15%) account for a large majority of the responses.

**Table 2:** What are the common barriers for training for energy efficiency in your organisation?

Barriers	Responses
Non-environmental friendly work procedures	2
Differences in competences of trainees	2
Not enough facilities for training	3
Not enough interest in the field	3
Language and communication issues	5
Procedural barriers	5
Non-realistic & non-flexible timeframes for training	6
Inadequate number and quality of training programs	7
Lack of trained manpower/staff	8
Not enough experience and lack of expertise in energy efficiency technology	8
Cost of training for energy efficiency	9
Inadequate understanding of the importance of a skilled workforce	10
Financial concerns and insecurities about the future that hinder investments in the field	10
Resistance to change	11
Not adequate demand for energy efficiency buildings	13
The challenge of creating more demand for energy efficiency	13
Not enough and proper information & awareness	16
Not enough time for training	24
Financial/funding issues	26

As to the level of provision of energy efficiency training at a national level, 33.33% responded positively (“Adequate level of training”), while a majority of 56.25% provided negative feedback (“Poor level of training”). When asked about the level of provision of energy efficiency training to a non-qualified workforce, the responses were spread across “poor” (29.63%) and “fair” (25.93%) ratings. The usefulness of the available training in promoting energy efficient interventions was judged positively by 20.83% of the respondents, while a majority of 54.17% replied negatively. Further to that, most respondents (61.90%) have been involved in knowledge and experience sharing in their organisation. As to the respondents’ views about the potential impact of training for energy efficiency, participants responded with high percentages, indicating that the value of training for energy efficiency affects not solely the construction sector (79.17%), but also the environment (85.42%), society (64.58%), and the economy (64.58%). When asked about the level of support to Diversity and Inclusion in energy efficiency, most respondents chose the “I do not know/I am not sure” option (31.71%).

Participants were then asked about the level of training for energy efficiency available at a European level to elicit their level of awareness. When asked whether they thought that the importance of energy efficiency training is being taken into consideration adequately, 43.75% responded positively, and 37.50% negatively. When asked about the BUILD UP Skills initiative to infer their level of awareness about energy efficiency training in Europe, 52.17% confirmed awareness of the initiative. Furthermore, the majority of those who knew about the initiative suggested it was useful (48.84%), while 57.50% suggested that initiatives like BUILD UP Skills should play a more important role in the European training landscape. With regards to recommendations to enhance training & skill development programs in the construction industry, most respondents (61.70%) selected “Make sure training has a significant practical contribution for those involved”, as reported in Table 3.

**Table 3:** What are your recommendations to enhance training & skill development programs in the construction industry?

Recommendations	Responses
Other	1
Have a sense of responsibility for the future impact of the training	10
Make sure certain parts of training are made core elements of curricula	12
Build up a database of companies involved in training	12
Demand more ambitious results	12
Establish support for funding initiatives that support training	14
Update relevant policies	15
Training take place in specific periods	15
Be supportive of any initiatives that promote awareness in the field	15
Make sure training and educational programs involved in energy efficiency are integrated in national frameworks	16
Make sure there is recognition/qualifications for the training undertaken	17
Make sure there are mandatory courses for construction workers	19
Make sure all parties and stakeholders involved are integrated in the process of developing training programs, from the start	21
Adequate promotion of training	22
Raise awareness for the need for training in energy efficiency	25
Make sure training is flexible and adjusts to the needs of those who undertake it	25
Make sure training has a significant practical contribution for those involved	29

When asked about the level of knowledge and experience sharing in the respondents’ organisations, a majority rated this as “Good”, with only 9.09% of participants suggesting “It is in a poor state”. When prompted to formulate recommendations to enhance training and skill development, most respondents referred to adaptable / tailored and flexible training (62.50%), as illustrated in Table 4

**Table 4:** What are your recommendations to enhance training and skill development programs in your organisation?

Recommendations	Responses
Other	2
Have a sense of responsibility for the future impact of the training	8
Establish support for funding initiatives that support training	10
Make sure certain parts of training are made core elements of curricula	11
Demand more ambitious results	12
Training take place in specific periods	15
Make sure there is recognition/qualifications for the training undertaken	15
Make sure there are mandatory courses for construction workers	17
Make sure all parties and stakeholders involved are integrated in the process of developing training programs, from the start	20
Make sure training has a significant practical contribution for those involved	21
Adequate promotion of training	24
Raise awareness for the need for training in energy efficiency	24
Make sure training is flexible and adjusts to the needs of those who undertake it	30

Concerning the scale of impact of energy efficiency training, most respondents reported that impact was felt at a local (52.08%) as well as national level (43.75%). When asked about whether they had received any training concerning energy efficiency in the construction sector, a larger sample of the participants confirmed that they had indeed received training (65.85%), while stating that this should be scaled up across other organisations and the wider industry (52.17%).

Furthermore, the training of trainers in energy efficiency programs was perceived as efficient and adequate (61.90%) by participants. The frequency and duration of the training that participants had been involved with was felt appropriate (59.52%), while the form of training provision involved in-person training via classes (62.96%), with an extensive use of “handouts, best practice guides” (66.67% of respondents), and online & video training (53.66%).

As to any financial implications of training, 50% of the respondents reported the “Difficulty in finding and training the required workforce”.

## Interview results

Twenty-eight interviewees took part in the interviewing process. The relevant nodes from the NVivo analysis are shown in Table 5.

**Table 5:** Nodes and subnodes, as used in NVivo

NODE 1	Re-usable best practice knowledge
SUBNODE 1	Barriers
SUBNODE 2	Market challenges & strategies
SUBNODE 3	Importance for energy efficiency skills
SUBNODE 4	BUILD UP Skills & relevant Programs
NODE 2	Industry-wide shared vision
SUBNODE 1	Barriers
SUBNODE 2	Market challenges & strategies
SUBNODE 3	Contribution to environmental awareness
SUBNODE 4	Knowledge & experience sharing
SUBNODE 5	Contribution to vision of long-term employment
NODE 3	Training in the energy efficiency domain
SUBNODE 1	Barriers
SUBNODE 2	Market challenges & strategies
SUBNODE 3	Training material
SUBNODE 4	Previous Knowledge, informal learning & training being integrated
SUBNODE 5	Link between academic & vocational training
SUBNODE 6	Focus on training (quality)
SUBNODE 7	Training programs
NODE 4	Trained and skilled workforce in energy efficiency
SUBNODE 1	Barriers
SUBNODE 2	Market challenges & strategies
SUBNODE 3	What can be done to increase demand
SUBNODE 4	Demand
SUBNODE 5	Skills that are needed
NODE 5	Legislative frameworks, policies, and government incentives
SUBNODE 1	Barriers
SUBNODE 2	Market challenges & strategies
SUBNODE 3	Qualification
SUBNODE 4	Integration of training in national strategies
SUBNODE 5	Integration of training in legislations and policies

First, interviewees were asked to elaborate on how training and skill development in the construction sector increase practitioners’ environmental and energy efficiency awareness. Most respondents confirmed the role of training in promoting sustainable interventions across the supply chain. This is reflected by one interviewee response stating: “… I do think it is one of the most important things because what our societies lack is the awareness of sustainability, knowing what should be done to be sustainable and what solutions are available, and to explain the benefits of the solution from a technical perspective. Effective training is very important”. Another interviewee argued: “there is no awareness of environmental issues and because of that there is no interest in upgrading skills, which could equip people, equip specialists to address the issue, to do something about it”. Lastly another interviewee sustained: “So raising awareness of how much we contribute to environmental issues. That is the second thing, the regulations.

Next, interviewees were asked to elaborate on barriers faced by the industry. Several barriers were mentioned, with the most frequent being: (a) time, (b) training and knowledge not being sufficient, (c) quality of training, (d) state of industry and issues of coordination, (e) legislation and regulation issues, as well as (f) motivation and incentives.

Interviewees were then asked to offer their insight on what can be done to increase demand for energy efficiency, in the construction sector. To this question, various suggestions emerged, which also included proposed solutions to several barriers highlighted above. One interviewee highlighted the challenge at hand, by highlighting the importance of legislation, stating: “ In my opinion, to increase the demand for energy efficiency in the construction sector there should be new and adequate government legislation and incentives to aid construction companies and homeowners willing to implement energy efficient methods for buildings.”

When asked about the current state of knowledge and experience sharing, with regards to energy efficiency in their organisation, overall, most interviewees sustained that the situation could overall be described as satisfactory, with one interviewee mentioning: “Knowledge and experience of energy efficiency in buildings in our organization is high as we work intensively in the field. Additionally, our experts take part in many experience and knowledge events and constantly improve their own qualifications”. Regarding aspects that can be improved, a variety of suggestions were made, such as (a) investment in technologies for energy efficiency, (b) awareness, (c) use of shared drives, (d) information sharing, (e) more interest in costumers, (f) deeper connections with the construction sector, (h) incorporate training in undergraduate studies, (i) legislation more efficiently targeting energy efficiency training, (j) lowering cost of production, (k) improving the comfort of workers, (l) working together with the network around, (m) voluntary energy efficiency agreements, (n) continuous professional development, (o) clear certifications and standardisations, (p) training bank representatives, and (q) using the knowledge of energy auditors.

However, when asked to comment on knowledge and experience sharing outside their organisation, most respondents reported clear limitations that resonate with the problematic fragmentation of the industry, and the dissonance in priorities and shared values, highlighting the barriers listed earlier. As reflected by one interviewee comment: “There is a lot of fragmentation in experience sharing.” Another interviewee argued: “At the moment the state of knowledge and experience sharing, with regards to energy efficiency, in the industry is very poor. Most companies lack trained manpower/staff, time, and money to provide adequate training to their workforce. I think that this can be improved by imposing new government legislation, providing adequate funding and incentives which will in turn create more demand for energy efficiency in the industry and will greatly increase the interest in the field”. Lastly, one interviewee mentioned, while referring to companies: “I think they might not be incredibly enthusiastic of sharing too much about their own projects with competitors […] they wouldn't do, and they probably put first the interest of their company in terms of profit rather than actually the environmental perspective.” Concerning aspects that can be improved, a variety of suggestions were made, such as (a) increased collaborative and shared space between project groups, (b) practical training, (c) new government legislations, (d) adequate funding, (e) incentives, (f) making sure that energy efficiency is actually implemented and not only discussed about, (g) clear standardisation, (h) SMEs to increase their knowledge and their employees’ background with specific training, (i) increase in demand for energy efficiency, (j) increase awareness of investors.

Interviewees were also asked to give their opinion on the level of demand for energy efficiency training and what they thought will happen in the foreseeable future. Overall, a positive outlook was presented, but with significant room for improvement, with one interviewee quoting … “it should increase rapidly with the enforcement of nZEB requirements for new buildings and deep renovation minimum energy performance requirements”. This was confirmed by another interviewee from a medium-sized organisation: “Today, every company and client in our surrounding is aware of its importance and demand is out there”.

Interviewees were then asked to comment on whether the importance of energy efficiency skills in the construction sector is being taken into consideration adequately in their field. To this question, responses were leaning more towards a negative perception, as reflected by one interviewee response: “When it comes to policy makers and advisers, yes. When it comes to companies themselves, many of the companies are holding off. When we look at training infrastructure, we are ready but when it comes to the raise in demand, the demand for training is not growing/developing as quick as we think it should.”

With regards to whether energy efficiency in the construction sector contributes to a vision of long-term employment, most interviewees suggested that, indeed, it does, with one interviewee highlighting: “Yes, because we must move from what we are doing now to a better world. We are in a critical situation relating to climate change and covid. Thinking about energy efficiency and sustainability could bring on long-term endurable opportunity for employment for designers, management, etc. There are a lot of opportunities relating to energy efficiency and buildings.”

As to how much of previous knowledge is considered in training programs for energy efficiency in the construction sector, as well as whether informal learning & training is being properly integrated, mixed answers were received as summarized by one interviewee from a training organisation: “…Most of the programmes start from zero and do not require previous knowledge. Informal learning and training are not properly integrated and is also not properly rewarded by companies.”

On a question about policies & legislation, and how effectively interviewees thought that they integrate training, very few stated that there is a clear link. One participant explained: “I think that they are not remarkably effective at the moment and definitely need to increase their efforts in integrating training programs.”

Interviewees were also asked to comment on how much training programs develop synergies between academic and vocational training. The replies indicate room for improvements, as highlighted by one respondent: “Well, I think there is a problem here, and now a generational change must probably take place simply in universities, to allow those who think and who care and act a little differently.” Another one argued: “I think that at the moment there is not good synergy between academic and vocational training. A secure way to strengthen the link is by providing more real-world practical experience and knowledge to trainees.”

With regards to market challenges that interviewees were able to identify, several challenges were mentioned, and financial issues were consistently mentioned. One participant observed: “Energy efficient solutions have higher investment price. Public sector as a client do not invest that much, in addition public sector is not demanding energy efficiency.”

When asked whether the insights of the training that they had been involved with were included in national strategies, the answers suggest potential scope for improvement. One participant explained: “We are training people that are already active in the field, through Continued Professional Development. If you develop those programmes in the right way, together with a good qualification scheme, then the regionals are usable within the rest of the education systems, so they can be used in vocational and academic education. If you do a proper job at skilling workers, you can use the same means to strengthen the regular education supply.”

Interviewees also praised the importance of initiatives such as BUILD UP Skills as they promote cross-fertilisation and sharing of learning materials. As one participant sustained: “They have been successful in creating an independent group of people working on the same topic and they are not doing it for their own government or for their national finances but for the European Union.”

Furthermore, interviewers were asked about whether training results lead to any formal (e.g., accredited) qualification and if these qualifications increase employability. To this question, most interviewees answered positively, as reflected in the following quote: “In my opinion training should result in formal qualification and it definitely increases employability chances as there are so few qualified workers in the sector.” Another one explained: “For energy efficiency, the accreditation is seldom for existing courses. Nevertheless, it is needed. The level of trust in official diplomas is crucial.”

Conversely, when asked to weigh on whether the focus placed on the training for energy efficiency is sufficient, a majority responded negatively. One participant argued: “Not yet. We need to focus more on energy efficiency, sustainability, and CO2 emissions. Again, we need clear requirements from legislation”.

Lastly, interviewees were asked to describe the skills that are needed in the new energy efficiency technologies in their field. Most replies pointed to skills that have to do with awareness, conceptual knowledge, and understanding skills, as being the most important. A holistic understanding of emerging energy efficiency needs in the construction sector was also highlighted. One participant suggested: “A general understanding of the whole process is beneficial. Knowing the concept of a sustainable building and understanding the bigger picture are essential. All in all, the whole working life in the construction sector is changing, and so workers need to be willing to change/upskill and evolve with the sector. People skills are essential, as collaboration is important in the sector.”

## Use cases results

A total of seventy (70) use cases were obtained using a template made widely available through the energy-education.com portal developed by the authors. Key insights from the use case analysis are given below:

- Use-case type: the template included three categories of use cases, namely Research & Development (35 use cases), Real-world applications (39 use cases), and Other (6 use cases).
- Building type: 67% of the gathered use cases relate to public buildings, 16% relate to domestic buildings, while 17% relate to industrial buildings.
- Project type: there is a balanced number of existing and new buildings (representing 50% of the gathered use cases), with an equal percentage focussing on renovation projects (50%).
- Targeted discipline: out of the gathered use cases, 31% focussed on Architectural design, 27% on Structural design, while mechanical engineering and facility management involved 14% and 17%, respectively.
- Lifecycle stage: the RIBA plan of work was used. 37% of the use cases focussed on the design stage, namely: stages 2 (Concept Design), 3 (Spatial Coordination) and 4 (Technical Design); 61% involved Stage 5 (Manufacturing and Construction), and 2% focussed on Stage 7 (Use Phase).
- Impact achieved: from the range of identified impacts, 20% of the use cases focused on optimisation of energy performance, increasing energy saving involved 33% of the use cases, and 14% can be attributed to reduction in carbon emissions, improving health and comfort involved 9% of the use cases.

It is worth noting that gathering quantitative evidence for the correlation between training and energy efficiency revealed to be a challenging undertaking. An interesting use-case was reported elaborating on the impact of BREEAM training on providing systematic means to improve energy efficiency of a hospital building, using the BREEAM concept of a notional building, concurrent with the compliant building regulations standards. The notional building is used to generate “indicative targets” for energy demand and consumption as well as the Target CO2 Emission Rate (TER). This process provides a formal and systematic method and benchmark to optimize the energy performance of a newly designed or refurbished building. The outcome of this benchmark is then translated into credits towards the BREEAM rating. This use case provided evidence of quantified energy reduction enabled by BREEAM training in excess of 35%.

**Fig. 2:**
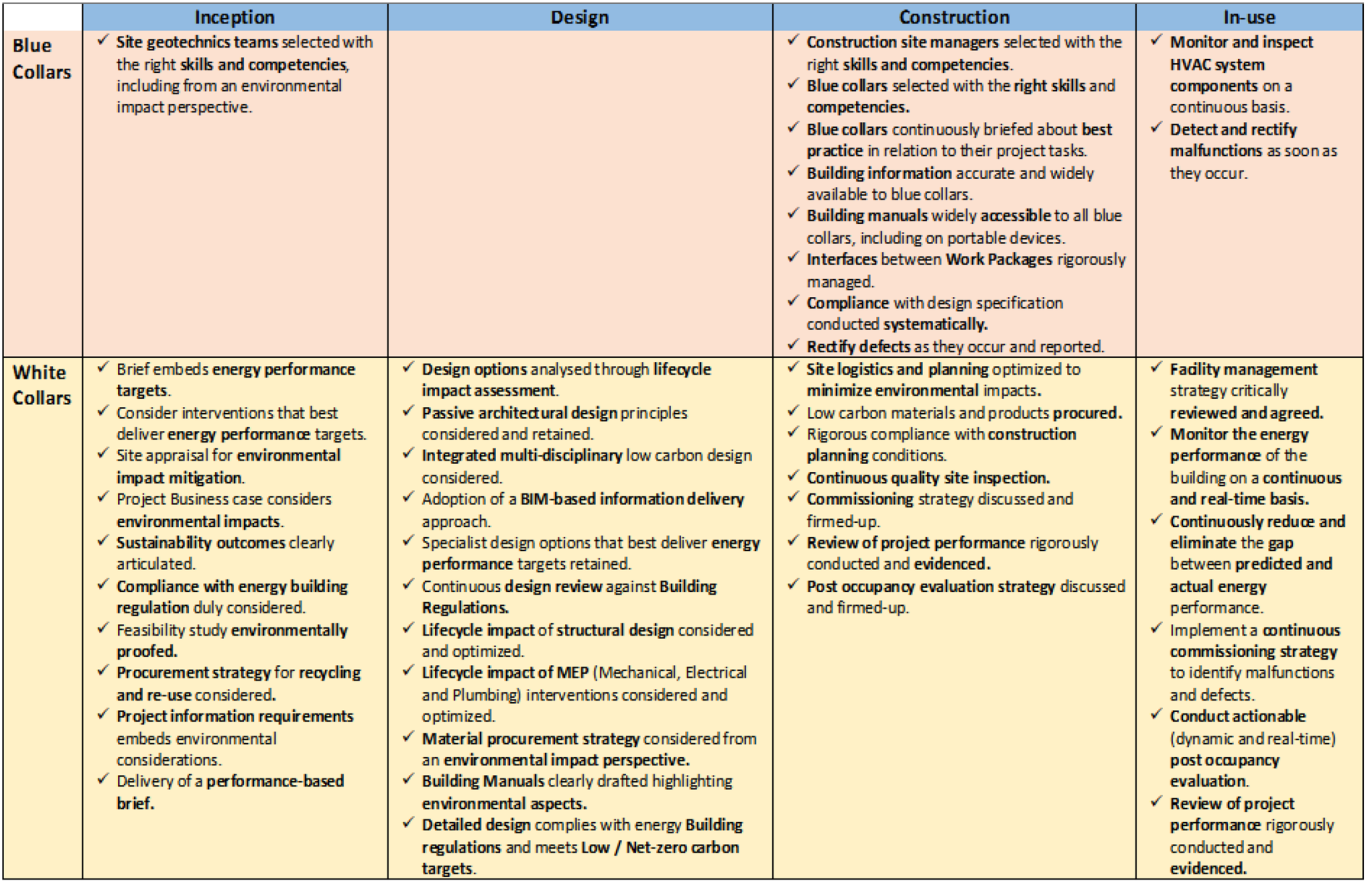
Measures conveyed through training with the potential to improve energy efficiency

Overall, the use-case analysis, augmented with other sources of evidence gathered from the literature and previous studies carried out by the authors (Rezgui and Miles 2010; Wilson and Rezgui 2014; Alhamami et al. 2020, Hodorog et al. 2020), provided an interesting ensemble of measures that prove to reduce energy consumption and carbon emissions by up to 50% (Barbero et al. 2022). These measures are summarized in Fig. 2 and structured by lifecycle (using four main stages, namely Inception, Design, Construction, and In-use) and user type (i.e., blue and white collars).

## Insights from the industry workshop

The purpose of the workshop was to share information and results from the three earlier instruments (i.e., Questionnaire, Interview, and use cases) and discuss and corroborate findings on energy efficiency and training in the construction sector with experts across Europe. A total of fifteen experts in training and education, from 8 European countries, took part in the workshop.

The five themes were formulated based on outcomes from the literature review as well as barriers highlighted by BUILD UP Skills (BUILD UP 2020), which were translated into the following questions:

- What is the state of awareness, access to information and dissemination of knowledge for energy efficiency in the Construction sector?
- What is the level of demand for skilled workforce in energy efficiency?
- What is the state of the training programs for energy efficiency currently available in the industry (in terms of scope, quality, content, cost, etc.)?
- What is the state of the sector in terms of shared values and coordination of stakeholders across the supply chain for energy efficiency?
- How efficient are legislative frameworks, policies, and government incentives?

This was translated into the following themes: (a) access to useful information, knowledge, and best practice guides in energy efficiency, (b) level of demand for skilled workforce in energy efficiency, (c) availability of energy efficiency training, (d) vision and values for energy efficiency in projects and across organisations, and (d) legislative framework and policies for energy efficiency.

With regards to access to useful information, knowledge, and best practice guides for energy efficient interventions, participants argued on the importance of industry demand for knowledge on energy efficiency. They then went on reflecting on the importance of creating the knowledge to be shared and argued that information/knowledge sharing and providing the right motivation is a cornerstone to the promotion of construction stakeholders’ engagement with sustainability. It was highlighted how best practice is often perceived as a double-edged sword as it needs to be understood and embedded within practices, as well as how there is a need for organisations to provide an environment supportive to knowledge sharing. One participant noted that: “… In my opinion, there is too much useful information, knowledge and best practice guides. […] There is an abundancy of materials, but the problem is those materials are not accessed or digested. A good way forward is to guide people that need to have access to knowledge that is relevant to them and that they really access it.” It was overall agreed that appropriately communicating the need for training is a very effective vehicle for imparting useful knowledge and sharing best practice. The significance of creating targeted training informed by market demand was also highlighted.

In line with the previous observations and concerning the lack of demand for a skilled workforce in energy efficiency, it became evident how there are factors in the construction sector which hinder reflective actions and capitalizing on lessons learnt, such as cost and time limitations. One participant argued that: “The high demand for workforce is overpowering the quality of the workforce, in a way”, Further to that, it was argued that the workforce is often not properly trained to be able to integrate workers in such endeavours. As one participant suggested: “There is an abundance of work and viscosity of workforce companies keep doing the same things, instead of innovating and adapting new business processes in order to tackle new markets”. Overall, participants noted the role of clients in promoting the demand for a skilled workforce and the need for a supportive legislative framework.

Concerning the level of availability of energy efficiency training, the workshop participants were in general agreement that current training is hindered by (a) a lack of understanding of the energy efficiency market demand and (b) industry skill needs, to inform the right training provision for the supply chain. Also, raising demand for such skills and understanding how to up-skill the workforce are factors that tend to be overlooked. It was suggested that blue-collar workers’ training is often not relevant / tailored to their needs and that more on-site training should be taking place. Also, the need for mobilisation of industry leaders, coupled with the importance of providing incentives for workers, in promoting adaptable training, was noted. One of the participants explained: “I do believe it is a common issue around Europe. […] The fact is that we are still not able to integrate the requirements for qualifications of both white and blue-collar workers in the procurement procedures at public and private levels.”

As to the lack of shared vision and values for energy efficiency across the supply chain, the overall message received from participants was that raising awareness for energy efficiency should be a priority. For this to happen, it was argued that a coordinated approach should take place, throughout the supply chain, where legislation and awareness campaigns might play a crucial role. One participant sustained: “It is a matter of establishing a common societal vision […] For that we need stronger awareness raising communication campaigns at various levels of an organisation”.

Lastly, regarding the inadequate policy landscape, including lack of government incentives, and as argued previously by participants, support from government and local authorities is critical. One participant commented on the matter that: “Without an adequate policy landscape, government initiatives or a government that is capable to understand what is needed, there is not much that we can do in particular parts of the construction sector, as well as other sectors”. It was observed that this is a European-wide issue and that, despite several initiatives across Europe, more collaboration and coordination between countries is needed, to develop a stronger policy landscape, while at the same time addressing country-specific barriers.

## Discussion

The aim of this study is to establish the relationship between training and energy efficiency. It is interesting to note that while initially the aim was to target both blue and white collars, in practice, only the primary sources of evidence gathered through use cases and the workshop involved insights concerning blue collars. In fact, while the questionnaire involved targeting contractors and sub-contractors via their federation, i.e., Federation of National Builders (FNB), the survey didn’t attract any response from this field of expertise, as illustrated in Table 1. A potential explanation, using our interpretive philosophical stance, and corroborated by related studies (Rezgui and Miles 2010; Wilson and Rezgui 2013), can be found in that (a) blue collars don’t have direct access to computers during their daily work to answer the questionnaire, (b) they don’t see direct benefit in answering the questionnaire outside work, i.e., during their social time, and (c) their employers (i.e., contractors and subcontractors) operate within very tight financial margins, so didn’t promote our consultation in their company, to not divert their workers from their tasks. We have, therefore, relied on the use cases and workshop to analyse and discuss the case of blue collars.

The study has overall explored the following themes:

1. Lack of systematic process to codify best practice into re-usable knowledge.
2. Lack of industry-wide shared vision.
3. Nature of the training available in the energy efficiency domain.
4. Level of reliance on a trained and skilled workforce in energy efficiency.
5. Efficiency of legislative frameworks, policies, and government incentives.

It has drawn on the quantitative and qualitative results and analysis of the data gathered from the above consultation, to answer the three posited research questions.

It is interesting to note that producing the quantitative evidence to correlate training with energy efficiency revealed challenging, as this information is sparse and often incomplete, as corroborated by related studies (Petri et al. 2014; Hodorog et al. 2020). The study also involves several limitations summarised below:

- The lack of representation of blue collars in the gathered primary sources of evidence.
- The limited size of the questionnaire sample (n = 52).
- The lack of quantitative sources of evidence to elicit the relationship between training and energy efficiency.

As elaborated earlier, the workshop was structured around 5 themes that were informed by the literature, including Build Up skills (BUILD UP 2020). Following the insightful inputs and approval, by the workshop participants, of the selected 5 themes, the authors used these themes to structure the discussion section, corroborated with facts from the other qualitative and quantitative sources of evidence gathered through the other instruments. In sum, the gathered evidence point to the following deficiencies discussed below: (a) lack of a systematic process to codify best practice into re-usable knowledge; (b) lack of an industry-wide shared vision on means to promote and implement energy efficiency measures on projects; (c) the nature of the training available in the energy efficiency domain; (d) level of reliance on a trained workforce; and (e) efficiency of legislative frameworks, policies, and government incentives. These are discussed in turn below using a triangulation approach informed by the above research instruments.

The importance of awareness and access to training has been highlighted as a significant factor. Lack of training for energy efficiency is highlighted as a barrier (Shapiro 2016), and its importance is recognised on a global level (Aerschot et al. 2009).

When it comes to an industry-wide shared vision, the sector presents a fragmented landscape (Rezgui and Miles 2011; Chaudhary et al. 2012), while more coordination is needed (Richards et al. 2016; Geros et al. 2006; Bosch González et al. 2013). With regards to the nature of training in energy efficiency, there is a need for continuous quality improvement (Milovanović et al. 2019). The need to tailor the training to the needs of the workforce has been highlighted in the literature (Levine et al.^2012^), as well as by the BUILD UP Skills initiative (European Commission 2016; Build Up 2020). Concerning matters of demand in the industry, a more focused demand for energy efficiency has been identified as a parameter which stimulates the need of training of workers (European Commission 2016). Also, BUILD UP Skills identified “low demand for energy efficient buildings and thus for the skills required to build them” (European Commission 2018), as a significant challenge. Lastly, legislative issues have been identified as critical, not only for future targets (Ministry of Energy 2016), but, also, when it comes to integrating changes into the fabric of the training landscape (Li and Yao 2009; European Commission 2018).

### lack of a systematic process to codify best practice into re-usable knowledge

It is interesting to note that capitalising on lessons learnt on projects remains a corporate exercise with little, with limited sharing of best practice on projects, as evidenced by our consultation. Overall, there is a general agreement, stemming from all gathered quantitative and qualitative data, that the current situation in the construction industry does not facilitate access to training and therefore awareness remains an issue to be addressed. In the questionnaire, 34.15% of participants lacked training for energy efficiency, while when asked if they had come across knowledge and experience sharing for energy efficiency, 61.90% replied with “yes”. The workshop pointed out to the fact that access is not the primary issue, but rather demand. This would put into perspective the priorities of the current needs of the construction industry and the high interconnectivity between different parameters. Overall, there seems to be a gap between the availability of training and the level of awareness from the construction workforce. In the questionnaires, 46.81% of participants argued for the need for further focus on the promotion of training, while 31.91% of participants suggested that more support towards training initiatives is needed. Similarly, 20 out of 28 interviewees suggested that the importance of training for energy efficiency is not being taken into consideration adequately. Several barriers were identified in the questionnaires such as “not enough and proper information and awareness”, while “Not-environmentally friendly procedures” and “not enough facilities of training” were also highlighted. Interviews similarly highlighted issues of awareness, and knowledge, with 5 out of 28 interviewees bringing them up when discussing market challenges. The corroborates findings from a related study (Wilson and Rezgui 2013). Barriers that were mentioned including “training & knowledge not sufficient”, and “access to training” were chosen by 8 interviewees. However, during the workshop, it was argued that it is not necessarily the lack of access, which is an issue but demand, which shapes the dynamics and sets the priorities of action. Conversely, the gathered use cases provided means to capture best practice cases and make these available across the company or beyond to promote knowledge sharing. However, this best practice gathering exercise is not often sustained (Wilson and Rezgui 2013). A need for better communication of the importance of training, a general improved guidance of the workforce, and more motivation for trainees were also deemed significant, as evidenced in a related study (Gantasala et al. 2022; Purwandani et al. 2021).

### Lack of an industry-wide shared vision

A lack of shared vision and values on many levels and across the supply chain were highlighted by the gathered data. In the questionnaires, 19.23% highlighted “incongruence of values between sectors and layers of stakeholders involved in the construction industry” as one of the barriers for training for energy efficiency. Discrepancy of knowledge and experience sharing between organisations and the industry, with 22 out of 28 interviewees and most questionnaire participants, respectively, pointing out a good state in the context of organisations, and 4 out of 28 interviewees and less questionnaire participants arguing this was the case in the industry. This was corroborated by workshop participants as well. It was also explained how raising awareness was a priority, and once again, demand was brough to the surface as a major key component. There was, however, a shared understanding of the importance that training for energy efficiency holds with regards to what could be argued or hoped that are shared values, across the supply chain, such as environmental, societal, and economic levels. A shift in perception when scaling up from the level of organisations to the entirety of the construction sector (less effective), with regards to knowledge and experience sharing was present. This became evident both in the questionnaires and in the interviews. The questionnaire data further seem to suggest that the effect of training for energy efficiency mostly hold an effect on a national and local level, so far. Moreover, in the interviews, 5 out of 28 participants highlighted issues of coordination between stakeholders in the construction industry. Also, when workshop participants were asked to comment on this, lack of shared vision and awareness were highlighted, and therefore the need for a better collaboration throughout the supply chain. Lastly, the gathered use cases demonstrated a pragmatic approach for capturing best practice in projects. However, these should transcend organisational boundaries and be shared across projects to be effective (Rezgui and Miles 2011; Wilson and Rezgui 2013).

### Nature of the training available in the energy efficiency domain

With regards to the data that addresses the quality and content of training programs in the field of energy efficiency in the construction sector, a varied outcome was uncovered. On one hand, there was a satisfaction in how the training was conducted (on a personal level), and on the other, suggestions that the training was not sufficient on a broader scale. In the questionnaires and interviews, most participants and interviewees alike were satisfied with the training they had personally received. Barriers that were mentioned, however, were: “Cost of training for energy efficiency” 17.31% & 23.08% “Inadequate number and quality of training programs” 13.46% & 11.54% “Non-realistic & non-flexible timeframes for training” 11.54% & 11.54%, as well “not enough time for training” 46.15% & 32.69%, and “inadequate understanding of the importance of a skilled workforce 19.23% & 21. 15%. Similar barriers emerged in the interviews, highlighting awareness issues, lack of skills in the field and education, and lack of time as the most significant barriers. When it comes to the nature and quality of training, the results highlight a need for improvement, which stems from a lack of an accurate interpretation of the needs of the workforce. Most participants in the questionnaire were satisfied with the quality of training they had received, and the same was argued by interviewees. However, once again, when arguing about the focus placed on training, interviewees sustained that it not sufficient (20 out of 28 interviewees). The workshop also pointed out that barriers such as “lack of time” and a “need for training to be as relevant as possible to the job” are significant. In the context of the questionnaires, several suggestions were made, in order to improve training, in the context of their organisations and the industry, respectively. Issues of adaptability and flexibility of training to meet the needs of the workforce and to motivate trainees, also emerged in the workshop. It is suggested that further research and more detailed attention should be placed in the field, to interpret any discrepancies, gaps, and room for improvement. It is also significant, to also to address and coordinate efforts on a European level.

### Level of reliance on a trained and skilled workforce in energy efficiency

The results have presented a complex landscape when it comes to the challenge of demand for a skilled workforce in energy efficiency. Demand to stimulate training for energy efficiency in the construction sector forms a key challenge, as it is highly dependent on the training landscape and overall context of the country, as well as stakeholders’ priorities. In the questionnaires, 25 identified “demand” as one of the barriers present in the construction sector, and when asked to suggest ways of increasing demand, awareness and legislations were at the top of suggestions. The interviewees, however, presented a different landscape with 10 of them replying that demand is insufficient, and 16 stating is sufficient. In the workshop, once again, the replies were mixed, for example in Finland it was suggested that high demand sometimes takes a toll on the quality of the workforce, while others argued how the role of clients, legislations and awareness is critical in further stimulating demand. Demand or issues around demand are perceived insufficiently addressed by several participants of the questionnaires. For example, responses to perceived barriers highlighted relevant issues with participants choosing 25.00% and 25.00% on “the challenge of creating more demand for energy efficiency”, 19.23% and 21.15% “inadequate understanding of the importance of a skilled workforce”, and 15.38% and 25.00% highlighted the “lack of trained manpower/staff”. The interviews responses with regards to demand were mixed, 10 argued that it was insufficient, while 16 suggested that it was sufficient. Both in the context of the interviews and the workshop, it was argued that demand in some contexts/ different countries etc. can be high, and that the importance of intensifying efforts towards awareness about the value of training is a significant key component.

### Efficiency of legislative frameworks, policies, and government incentives

The findings on the policy landscape, in general, seem to indicate that there is currently a gap that needs to be addressed. From the need to “Make sure training and educational programs involved in energy efficiency are integrated in national frameworks” (34.04%), and “update relevant policies” (31.91%), to “Procedural barriers” (9.62%) & (15.38%), “Lack of government incentives” (23.08%) & (30.77%), as well as “inadequate policies and legislations” (19.23%), the questionnaire data presents a need to strengthen efforts. Further support of this evidence was provided by the interviews. Interviewees highlighted legislation and regulation issues when asked about barriers (5 out of 28 interviewees). 16 out of 28 interviewees also commented on the need to further integrate training for energy efficiency in policies and legislation. Similarly, one of the workshop’s main messages was that across Europe and based on different contexts the link between policies and training for energy efficiency differs. However, there was a generalised understanding of a need to coordinate efforts across Europe, as well as of the fact that support from the governments is key towards changes in the field of training for energy efficiency. Results suggest there needs to be a more cohesive coordination of the sector, on a European level. “Interviewees commented on how effectively policies and legislation integrate training (e.g., the European Green Deal, which focuses on making EU’s economy sustainable and EU climate neutral by 2050). Workshop participants also argued on the importance of political power and governments to push for change, which seems to be a European-wide issue. Furthermore, there was a suggestion that similar projects which studied the role of legislations in the construction sector, in relation to training for energy efficiency, need to be open to promote sharing of best practice and relevant knowledge.

## Conclusion

Outcomes from the consultation reported in the previous sections point to several energy efficiency barriers, exacerbated by the fact that energy use and efficiency measures tend to focus mainly on the diffusion of efficient technologies, such as high energy performance construction products (e.g., facades) as well as renewable technologies, but less on energy management best practices, including training and education. In fact, investments in technology and upgrading equipment, as well as the introduction / adaptation of incentives and strengthening of the regulatory frameworks, generate improved efficiencies, but without adapted training the efficiency potential will not be attained.

Three research questions (RQ1, RQ2, and RQ3) are posited in this study. As to RQ1 (state of awareness, access to information and dissemination of knowledge for energy efficiency in the Construction sector), the study outcomes reveal a lack of a systematic process to codify best practice into re-usable knowledge in organisations, and even when in place, the knowledge stays within the organisation and is often not shared on projects. This can be explained by a lack of industry wide vision on energy efficiency supported by a robust legislative framework, policies, and government incentives. These tend to vary from country to country.

As to RQ2 (level of provision of energy efficiency training in the Construction sector), the study reveals a mixed landscape with a clear lack of understanding of market demand based on a lifecycle and supply chain segment. This corroborates findings from related studies (Backlund et al. 2012; Maier et al. 2019), that report: (a) a wide variety in the quality of training provision, (b) fall in apprenticeship completions due to challenging economic conditions, (c) reliance on a more flexible self-employed workforce due to uncertainty in the market, (d) Low training and development activity driven by the high number of self-employed tradesmen who often face an ‘earn or learn’ dilemma, (e) the transient nature of the workforce and the evolving training demand of the industry deterring employers from investing in staff training, (f) lack of career planning and the tendency to adopt a supplier as opposed to a demand driven model, (g) lack of strategic approach to Continuing Professional Development (CPD) and Continuing Craft Development across the industry.

As to RQ3 (does training translates into effective sustainable and energy efficient interventions?), while there is abundant qualitative evidence, the study points to a lack of a comprehensive and robust quantitative evidence for correlating training with energy efficiency. This can be attributed to the complex dynamic nature of buildings, and the lack of post-construction and post-occupancy evaluations within organisations, projects, and the wider industry (Wilson and Rezgui 2013).

Conversely, the study has highlighted-specific areas of improvement in the construction sector, regarding the link between training and energy efficiency, as described by the five themes of the study:

1. Lack of systematic process to codify best practice into re-usable knowledge.
2. Lack of industry-wide shared vision.
3. Nature of the training available in the energy efficiency domain.
4. Level of reliance on a trained and skilled workforce in energy efficiency.
5. Efficiency of legislative frameworks, policies, and government incentives.

By analysing the different themes, the researchers have carried out an in-depth investigation of the gaps, barriers, needs and drivers in the industry. As a result, this study presents a holistic picture of the causes of the current fragmentation in the construction sector, which will inform policy to effectively address the identified gaps.

This study has made several contributions to the existing body of knowledge, which we have summarized below:

- An in-depth and sector-wide study to understand the barriers and challenges in the training landscape in the European construction sector.
- A qualitative evidence base pointing to a positive relationship between training and energy efficiency in the Construction sector, corroborated with available quantitative and secondary sources of evidence.
- The need to develop instruments that can recognise the skills of blue and white collars as well as increasing the demand for energy efficiency skilled blue and white collars.

Future research may exclusively pursue and focus on quantitative evidence gathering to further substantiate the quantitative correlation between training and energy efficiency, while also providing a broader coverage of the supply chain, including blue collars, as well as the lifecycle dimension.

## Annex A: interview guide

See Table 6.

**Table 6:** Interview questions, categorised by theme

Theme	Interview questions
∙Lack of a systematic process to codify best practice into re-usable knowledge (lack of access to useful information, knowledge, and best practice guides for energy efficient interventions)	∙Q4. What barriers can you identify in the field of training for energy efficiency, in the construction sector? ∙Q9. In your opinion, is the importance for energy efficiency skills in the construction sector being taken into consideration adequately, in your field? ∙Q19. What market challenges can you identify, concerning demand & economic changes? Are there any strategies that have been identified as successful in dealing with these challenges? ∙Q21. In your opinion, have initiatives such as the BUILD UP Skills been successful and in what manner?
∙Level of reliance on a trained and skilled workforce in energy efficiency (lack of demand for skilled workforce in energy efficiency)	∙Q4. What barriers can you identify in the field of training for energy efficiency, in the construction sector? ∙Q5. What can be done, in your opinion, to increase demand for energy efficiency, in the construction sector? ∙Q8. Could you please give your opinion on the level of demand for energy efficiency training and what you think will happen in the foreseeable future? ∙Q12. Could you please describe the skills that are needed in the new energy efficiency technologies, in your field? ∙Q19. What market challenges can you identify, concerning demand & economic changes? Are there any strategies that have been identified as successful in dealing with these challenges?
∙Nature of the training available in the energy efficiency domain (Lack of availability, or inadequate, training programs (in terms of scope, quality, content, cost, etc.)	∙Q4. What barriers can you identify in the field of training for energy efficiency, in the construction sector? ∙Q10. Is the focus placed on training for energy efficiency sufficient? Please elaborate on your opinion? ∙Q11. Could you give any examples of other training programs in the construction industry that you believe are contributing to energy efficiency, in the construction sector? ∙Q14. How comprehensive is the training material for energy efficiency in the construction sector that you are familiar/involved with (and if you can elaborate on what that training is)? How can it be improved? ∙Q15. How much of previous knowledge is considered in training programs for energy efficiency in the construction sector? Is informal learning & training being properly integrated? ∙Q18. How much do training programs develop synergies between academic and vocational training? What could be done to further strengthen this link? ∙Q19. What market challenges can you identify, concerning demand & economic changes? Are there any strategies that have been identified as successful in dealing with these challenges?
∙Lack of an industry-wide shared vision (lack of shared vision and values for energy efficiency across the supply chain)	∙Q3. How does training and skill development in the construction sector contribute to the increasing need for environmental awareness, in our societies? ∙Q4. What barriers can you identify in the field of training for energy efficiency, in the construction sector? ∙Q6. What is the current state of knowledge and experience sharing, with regards to energy efficiency, in your organisation, in your opinion? What can be done to improve it? Are there any conflicting interests? ∙Q7. What is the current state of knowledge and experience sharing, with regards to energy efficiency, in the industry, in your opinion? What can be done to improve it? Are there any conflicting interests? ∙Q13. Does energy efficiency in the construction sector contribute to a vision of long-term employment? ∙Q19. What market challenges can you identify, concerning demand & economic changes? Are there any strategies that have been identified as successful in dealing with these challenges?
∙Efficiency of legislative frameworks, policies, and government incentives (Inadequate policy landscape, including lack of government incentives)	∙Q4. What barriers can you identify in the field of training for energy efficiency, in the construction sector? ∙Q16. Does completing training result in any formal (e.g., accredited) qualification? Do these qualifications increase employability? ∙Q17. With regards to policies & legislation, how effectively do you believe they integrate training? (e.g., the European Green Deal, which focuses on making EU’s economy sustainable and EU climate neutral by 2050) ∙Q19. What market challenges can you identify, concerning demand & economic changes? Are there any strategies that have been identified as successful in dealing with these challenges? ∙Q20. Have any aspects/insights of the training that you have been involved with been included into national strategies?

## Annex B: Questionnaire

See Table 7.

**Table 7:** Questionnaire questions, categorised by theme

Theme	Survey questions
∙Lack of a systematic process to codify best practice into re-usable knowledge (Lack of access to useful information, knowledge, and best practice guides for energy efficient interventions)	∙Q6. What are the common barriers for training for energy efficiency in your organisation? ∙Q7. What are the common barriers for training for energy efficiency in the industry?
	∙Q10. Are you aware of the BUILD UP Skills initiative? ∙Q11. In your opinion, was BUILD UP Skills, successful? ∙Q12. Should initiatives like BUILD UP Skills be undertaken in the future? ∙Q15. What are your recommendations to enhance training & skill development programs in your organisation? ∙Q16. What are your recommendations to enhance training & skill development programs in the construction industry? ∙Q19. Overall, is the focus placed on energy training for energy efficiency sufficient, in the construction sector, in your opinion? ∙Q20. Have you been involved with knowledge and experience sharing for energy efficiency in the construction sector? ∙Q23.Have you ever received any training concerning energy efficiency in the construction sector?
∙Level of reliance on a trained and skilled workforce in energy efficiency (Lack of demand for skilled workforce in energy efficiency)	∙Q6. What are the common barriers for training for energy efficiency in your organisation? ∙Q7. What are the common barriers for training for energy efficiency in the industry? ∙Q22. Could you please identify any issues linked with the financial implication of training (hidden transaction costs)? ∙Q27. In what manner does training for energy efficiency deal with retiring workforce, in the construction sector? ∙Q28. In what manner does training for energy efficiency deal with non-qualified workforce, in the construction sector? ∙Q29. In what manner does training for energy efficiency deal with next-generation workforce, in the construction sector? ∙Q30. In what manner does training for energy efficiency deal with next-generation workforce, in the construction sector?
∙Nature of the training available in the energy efficiency domain (Lack of availability, or inadequate, training programs (in terms of scope, quality, content, cost, etc.)	∙Q6. What are the common barriers for training for energy efficiency in your organisation? ∙Q7. What are the common barriers for training for energy efficiency in the industry? ∙Q15. What are your recommendations to enhance training & skill development programs in your organisation? ∙Q16. What are your recommendations to enhance training & skill development programs in the construction industry? ∙Q24. What type of material was used in the training program for energy efficiency in the construction sector that you have been involved with? ∙Q25. Was the training of trainers in programs of energy efficiency in the construction sector efficient & adequate, in your opinion? ∙Q26. Was the frequency and level of detail, including duration of the training that you have been involved with, appropriate?
∙Lack of an industry-wide shared vision (Lack of shared vision and values for energy efficiency across the supply chain)	∙Q6. What are the common barriers for training for energy efficiency in your organisation? ∙Q7. What are the common barriers for training for energy efficiency in the industry? ∙Q8. What is the current state of knowledge and experience sharing with regards to energy efficiency in your organisation, in your opinion? ∙Q9. What is the current state of knowledge and experience sharing in the industry, with regards to energy efficiency, in your opinion? ∙Q18. On what level does the positive impact/results of training in the construction sector for energy efficiency becomes more evident, in your opinion?
∙Efficiency of legislative frameworks, policies, and government incentives (Inadequate policy landscape, including lack of government incentives)	∙Q6. What are the common barriers for training for energy efficiency in your organisation? ∙Q7. What are the common barriers for training for energy efficiency in the industry? ∙Q13. In your opinion, is the importance of energy efficiency training being taken into consideration adequately by the construction industry, on a European level? ∙Q14. In your opinion, is the importance of energy efficiency training being taken into consideration adequately by the construction industry, on a national level?
